# Biomarkers and potential subtypes of acute mountain sickness: A state-of-the-science review

**DOI:** 10.1016/j.redox.2026.104260

**Published:** 2026-06-16

**Authors:** Johannes Burtscher, Roxana Ehlers, Nicolas Bourdillon, Evelyn R. Pircher Nöckler, Hannes Gatterer, Katharina Hüfner, Johanna M. Gostner

**Affiliations:** a Department of Psychiatry, Psychotherapy, Psychosomatics and Medical Psychology, University Hospital for Psychiatry II, Medical University of Innsbruck, Innsbruck, 6020, Austria; b Institute of Sport Sciences ISSUL, University of Lausanne, Lausanne, 1015, Switzerland; c Teaching and Research Unit in Physical Education and Sport (UER EPS), University of Teacher Education (HEP-VD), Lausanne, 1015, Switzerland; d Institute of Mountain Emergency Medicine, Eurac Research, Bolzano, 39100, Italy; e Institute for Sports Medicine, Alpine Medicine and Health Tourism (ISAG), UMIT TIROL-Private University for Health Sciences and Health Technology, Hall in Tirol, 6060, Austria; f Institute of Medical Biochemistry, Medical University of Innsbruck, Innsbruck, 6020, Austria

**Keywords:** High altitude illnesses, Acclimatization, Hypoxia responses, Oxidative stress, Hypoxia-inducible factor, Lake Louise score

## Abstract

Exposure to high altitude can cause acute mountain sickness (AMS) characterized by headache, gastrointestinal symptoms, dizziness and fatigue, which may progress to life-threatening conditions like high-altitude cerebral edema (HACE). Although the pathophysiology and development of AMS have been intensely investigated, the risk factors and underlying mechanisms remain incompletely understood. In addition, objective criteria for the diagnosis of AMS and reliable biomarkers are missing. Here we provide an overview of the molecular and pathophysiological foundations of AMS and review which potential biomarkers have been suggested and in combination with which physiological and psychological correlates of AMS they could improve diagnosis. Moreover, we point out current knowledge gaps and discuss which future research is required to enable AMS diagnosis based on more objective biomedical and psychophysical criteria. Emphasizing the apparent heterogeneity in AMS pathogenesis, we support the classification of different AMS subtypes according to etiological parameters. We propose the hypothesis that both insufficient and excessive hypoxia responses can cause AMS and that the differentiation according to these divergent mechanisms might allow the identification of AMS subtypes that can be characterized better using biomarkers and correlates of AMS.

## Graphical abstract

**Figure:**
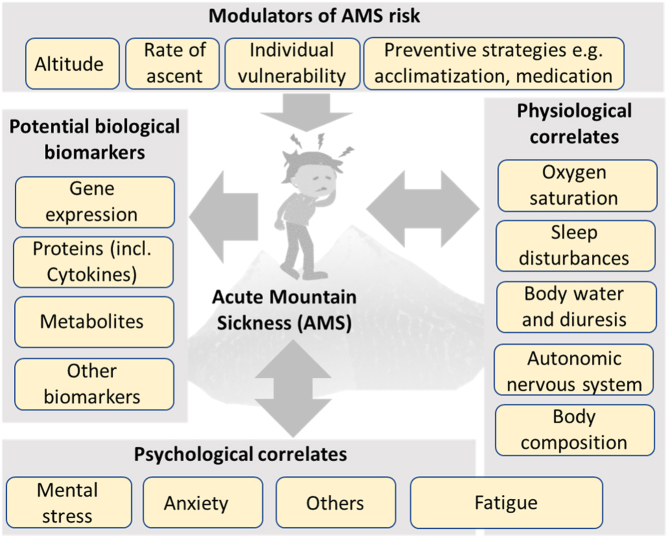

## 1 Background: acute mountain sickness

Acute mountain sickness (AMS) is a common syndrome experienced during exposure to hypoxia, a condition characteristic for high-altitude environments. Altitudes higher than about 2500 m can trigger AMS in unacclimatized people, although vulnerable individuals may develop AMS at lower altitudes [1]. AMS occurs normally during the first 5 days, but usually not before 4 h at high altitude and symptoms frequently peak after the first night spent at a new altitude. The incidence of AMS correlates well with the hypoxia severity and the rate at which the partial pressure of oxygen (e.g., hypobaric hypoxia at altitude; i.e., speed of ascent) or concentration of oxygen (normobaric hypoxia) declines [1,2]. In most cases, AMS is transient and resolves with acclimatization, descent from high-altitude locations or oxygen supplementation. It may, however, be involved in the development of high-altitude cerebral edema (HACE), a potentially fatal altitude disease, as AMS and HACE share some, but not all, pathological processes [3,4]. A history of AMS (AMS susceptibility [5]) increases the risk for the development of future AMS, and a history of migraine and/or a small ventilatory response to hypoxia may represent minor risk factors as well [1,6]. Potential risk factors like age, sex, body mass index, fitness and training status, alcohol consumption and cigarette smoking have previously been reported not to significantly influence the development of AMS [7], although reports are not consistent and some do link certain factors such as body mass index [8] to an increased risk of AMS. Likewise, reports on a higher vulnerability of women compared to men remain controversial [5] and the influence of age on AMS risk is not fully understood, although older age (>50 or 60 years) may be protective [5,9,10] and the prevalence of AMS in children has been reported to be delayed, with significantly lower risk for children to develop AMS on day 1, but not day 2, at 3500 m [11]. A more recent meta-analysis evaluating 17 studies on AMS in different age-groups, however, concluded that there is no statistically significant association between age and AMS [12]. Overall, individual demographic, genetic or health related risk factors for AMS are still debated. However, due to differing protocols and study contexts, the resulting heterogeneity may limit comparability and complicate interpretation. In addition, it has been suggested that obesity [10] and lung diseases may be additional risk factors, but in general most pre-existing medical conditions appear not to substantially modify AMS risk [1].

At the same time, it is well established that the risk to develop AMS is greatly reduced with a slow rate of ascent, pre-acclimatization or medication. Increasing altitude (or hypoxia severity) elevates the risk of AMS, from less than 10% at 2500 m to more than 40% above 4500 m, when ascending slowly [2]. Fast ascent (e.g., by cable car or aircrafts) increases the risk of AMS dramatically, with one study reporting a rate of 84% of unacclimatized adult tourists developing AMS, when flying to 3740 m from low altitude [13]. Previous exposure to hypoxia (acclimation) or altitude (acclimatization) are highly protective, but time demanding. According to a model of accumulating altitude exposure, every 1 km d (calculated as altitude elevation (km) multiplied by the number of days (d) at that altitude) increase in accumulated altitude exposure reduces the risk for AMS by 41.3% when ascending to 4000 m [14]. Pre-acclimatization to (simulated) altitudes of 2200 to 5000 m for a total of >200 h appears to prevent AMS entirely for altitudes up to 8000 m [15]. Remote ischemic preconditioning may also attenuate AMS symptoms initially but appears to rather delay and not prevent AMS [16]. A recent study [17] suggests that the combination of ischemic preconditioning with pharmacological prevention (acetazolamide) may be a highly effective strategy to prevent AMS. This combination reduced AMS prevalence in a sample of 250 subjects exposed to simulated altitude of 4000 m from 26% to 6% [17]. Intermittent hypoxia preconditioning can reduce AMS [18] and attenuate hypoxia-induced damage to the brain or damage indicated by hematological parameters [19].

**Fig. 1:**
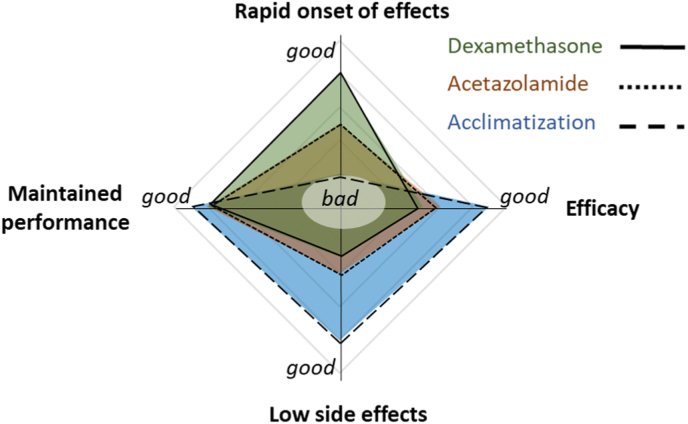
**Comparison of preventive strategies for acute mountain sickness (AMS).***Non-pharmacological acclimatization strategies can be very efficacious for preventing AMS and minimizing physical and physiological performance decrements at high altitude, its efficacy improving with increasing pre-exposure time. Many hours of hypoxia/high-altitude exposure are required for optimal acclimatization: depending on the target altitude, many days to weeks with exposures close to the target altitude can be necessary. Acetazolamide represents a pharmacological tool to promote acclimatization, dexamethasone is particularly useful for rapid ascents without acclimatization. The figure was created with MS powerpoint.*

Acetazolamide (at the doses 250 mg, 500 mg and 750 mg), which is commonly used for AMS prevention, reduced the odds ratio to develop AMS by about 0.36 (confidence intervals 0.28 to 0.46) in healthy lowlanders older than 16 years ascending to >3000 m [20]. Current recommendations for medication to prevent AMS (for people with known AMS vulnerability) are 125 mg acetazolamide every 12 h or 2 mg dexamethasone every 6 h; to treat AMS, 250 mg acetazolamide every 12 h or 4 mg dexamethasone every 6 h are recommended, the latter being only considered for special situations, e.g., rescue missions [1,21]. Acetazolamide is a carbonic anhydrase inhibitor and supports acclimatization by inducing a mild metabolic acidosis that stimulates ventilation and improves oxygenation, with relatively low side effects (at the recommended dosages paresthesia can result, while at higher dosages polyuria and taste disturbances occur [22]). While long-term use of dexamethasone (like other corticosteroids) can be associated with unwanted side effects (e.g., adrenal suppression, psychiatric adverse effects), no serious side effects have been described for its use against AMS and its rapid onset of effects makes it particularly useful, when rapid ascents without acclimatization are necessary [23]. Symptoms of AMS can also be treated with acetaminophen or aspirin (headache) or antiemetics (nausea). A comparison of prevention strategies of AMS is shown in Fig. 1, the role of dexamethasone as a rapid onset medication for AMS prevention and treatment has recently been reviewed [23].

Cellular and systemic responses to hypoxia are involved in the development of AMS. Therefore, tolerance to hypoxia appears to be an important protective factor against AMS. Among the most obvious potential biomarkers for general hypoxia responses are hypoxia inducible factors (HIFs), heat shock proteins and nitric oxide (NO) [24]. By themselves, these biomarkers are clearly not specific enough to provide clinically useful information on AMS. As of today, no biomarkers for AMS have entered clinical routine and AMS diagnosis is based primarily on self-reported symptoms. Therefore, the identification of specific individual biomarkers or biomarker combinations should be goals of future research on the molecular characterization and diagnosis of AMS. The aim of the present review is to summarize the progress made on biomarker discovery for AMS and derive important next steps for the development of more objective diagnostic and predictive criteria for AMS, including a classification of potential subtypes of AMS.

## 2 Etiology and diagnosis of acute mountain sickness

Despite much research and increasing knowledge on the pathophysiology and development of AMS, risk factors and underlying mechanisms remain poorly understood. Theories (previously summarized in more detail [[25], [26], [27]]) trying to explain the development of AMS include increases in intracranial pressure linked to changes in cerebral blood flow and cerebral vasodilation. Moreover, blood-brain barrier disruption, oxidative stress-mediated activation of the trigeminovascular system may also result in headache and/or AMS. Inflammatory mechanisms, potentially in combination with inadequate venous drainage, are potential further important components of the pathophysiology of AMS.

Decreasing arterial oxygen saturation (as a result of ambient hypoxia and/or exercise, and low ventilatory response at altitude) has been shown to predict higher AMS risk [28,29], although by itself it does not accurately reflect AMS risk [30]. In addition, hypohydration at altitude tended to influence AMS score in young male lowlanders [31]. Moreover, fluid retention was reported to influence AMS development [[32], [33], [34]], even though contrasting reports exist [35]. Recent reports are inconsistent on changes in average blood-pressure values in AMS, with some authors describing increases [36] and others not [37].

The metabolic challenge of hypoxia and specific cellular hypoxia responses, including for example increased expression of vascular endothelial growth factor (VEGF), are also involved in AMS pathogenesis [38]. Increased levels of VEGF led to hyperpermeability of blood vessels and, notably, of the blood-brain barrier in animal models [[39], [40], [41]]. In humans, serum and plasma VEGF concentrations were higher in AMS susceptible mountaineers [38,42]. Excessive VEGF activity may thus transiently compromise vessel integrity and permeability, thereby contributing to AMS development.

The diagnosis of AMS relies predominantly on self-reported rating systems, primarily the Lake Louise Acute Mountain Sickness Score (LLS), the most commonly used self-questionnaire-based rating system to diagnose AMS [43,44]. The primary symptom required for LLS-based AMS diagnosis is headache related to a recent exposure to altitude. Other symptoms evaluated with the LLS are gastrointestinal symptoms (reduced appetite and nausea/vomiting), dizziness and lassitude/fatigue [43]. It has, however, been argued that headache should not be a required symptom for AMS diagnosis, based on the observation that AMS-like symptoms attributable to high-altitude can occur in the absence of headache [45,46].

Other instruments to diagnose AMS include the Environmental Symptoms Questionnaire (ESQ) [47] or its shortened version [48], the AMS-Cerebral Score (AMS-C) derived from the ESQ, the Chinese AMS Score (CAS) [49], the Hackett Clinical Score, visual analog scales for the Feeling of Sickness at Altitude Score [50], and the Clinical Functional Score (CFS), as reviewed by Meier and colleagues [5]. Visual analogue scales are also frequently used in combination with other tools for AMS diagnosis, when headaches are being evaluated.

Despite the existence of these diagnostic tools, reliable and robust diagnostic criteria, such as clinically used biomarker systems, for AMS are missing. Overall, a lack of a clear mechanistic understanding of AMS etiology and of objective biomarkers impede diagnosis and the development of new efficient treatment options. The diverse physiological responses and biological pathways implicated in AMS, in addition to the heterogeneous symptomatic manifestations suggest that different subtypes of AMS exist [51]. This, to some degree, could explain inconsistent findings on AMS risk factors, heterogeneous reports on biomarker validities and why some individuals do not profit from medications that facilitate acclimatization. The categorization of AMS in more homogenous subtypes therefore might enable the development of more valid and reliable biomarkers, with considerable implications for diagnosis and treatment.

## 3 Hypoxia sensing and acute hypoxia responses

Reduced inspired oxygen, either due to a low partial pressure of oxygen in the ambient air (hypobaric hypoxia, such as occurring at high altitude) or reduced oxygen concentration in the inspired air (normobaric hypoxia, e.g., occurring in normobaric hypoxic chambers) leads to hypoxemia, which is considered the causative factor for AMS [52]. An immediate physiological response to reduced inspired oxygen is increased breathing (hyperventilation) and alveolar minute ventilation [53,54]. This response, however, does not only increase oxygen uptake, but also results in more carbon dioxide being breathed out, lowering the partial pressure of carbon dioxide in the alveoli and in arterial blood and causing respiratory alkalosis (an increase of pH due lower levels of carbon dioxide in the blood). As a resulting compensatory mechanism to re-establish a normal pH, bicarbonate is excreted from the kidneys [55]. The efficacy of using inhibitors of carbonic anhydrases as a therapeutic approach to prevent AMS, would suggest this compensatory process to be involved in the development of AMS. Carbonic anhydrase inhibitors that are indeed used in the prevention of AMS, such as acetazolamide, catalyze the production of bicarbonate.

Both hypoxemia and reduced arterial carbon dioxide are sensed by chemosensors. Especially the carotid bodies signal low partial pressures of oxygen to respiratory brain stem centers and modulate the autonomic responses [56]. Together with the NO-mediated vasodilation of most blood vessels in hypoxia, these effects lead to cardiorespiratory outcomes like increased heart rate, cardiac output and cerebral blood flow. Importantly, hypoxia in the lungs leads to pulmonary vasoconstriction, which is involved in the pathogenesis of the potentially fatal high-altitude pulmonary edema (HAPE) [25]. While peripheral vasodilation largely counteracts the sympathetically-mediated increase of blood pressure in laboratory settings of hypoxia, exposure to real altitude is frequently associated with higher blood pressure [57].

At the cellular level, hypoxia induces a wide array of molecular and metabolic responses [58,59]. The reduced oxygen availability directly impairs oxygen-dependent enzymatic reactions and oxidative phosphorylation, in which adenosine triphosphate (ATP), the major cellular energy currency, is produced. Moreover, hypoxia-induced changes in redox homeostasis and cellular metabolism, hinging for example on the hypoxia-sensitive redox couple nicotinamide dinucleotide (NAD, oxidized and reduced form), occur instantly [60]. Although hypoxia in cell cultures and laboratory conditions does not increase oxidative stress, it can be assumed that altitude exposure in humans does [61,62], leading to oxidation and nitration of various cellular components across many tissues [63] and may compromise for example the integrity of the blood-brain barrier. Oxidative stress is thought to play an important role in the pathogenesis of AMS [64]. Supporting this notion, plasma lipid peroxidation has been shown to be correlated with indicators of AMS, such as arterial and peripheral oxygen saturation and Lake Louise score [65] also independent of blood-brain barrier disruption [66].

Some of the affected oxygen-dependent enzymes inhibited in hypoxia are HIF-prolyl hydroxylase domain enzymes (PHDs). In normoxia (and presence of alpha-ketoglutarate and ferrous iron), PHDs hydroxylate the alpha-subunits of a family of transcription factors, HIFs, which leads to their degradation [59,67]. In hypoxia, HIF alpha-subunits stabilize, bind HIF1-beta subunits and regulate gene transcription, by interacting with hypoxia response elements (HRE). HIF1 and HIF2 are major coordinators of the transcriptional response to hypoxia and coordinate diverse hypoxia responses, like the upregulation of glycolytic enzymes and proteins involved in the remodeling and new formation of blood vessels, such as for example VEGF, a likely mediator of AMS-development as discussed above. Both HIF variants have different temporal kinetics and functions, with HIF1 being transiently upregulated and involved more in acute hypoxia responses, while HIF2 upregulation is slower but longer-lasting [68].

Next to HIFs, also other transcription factors are induced by hypoxia and often interact with HIFs. Among them are notably master regulators of antioxidant-responses, such as nuclear factor erythroid-2 related factor 2 (Nrf-2) [67], and of inflammatory responses, e.g., nuclear factor κB (NF-κB) [69]. In addition, hypoxia can trigger translational, post-translational and overall proteostasis-protecting responses, as previously reviewed [58]. These mechanisms include the initiation of the unfolded protein response following endoplasmic reticulum stress and reduced mammalian target of rapamycin complex 1 (mTORC1) activity, both downregulating translation [58].

While it has traditionally been assumed that too small hypoxia responses (in particular an insufficient hypoxic ventilatory response) cause AMS, it is also possible that an overshooting response is the main culprit [27]; e.g., increased cerebral blood flow and leakage due to overactivation of the HIF-VEGF axis, or excessive inflammatory responses. In any case, inadequate hypoxia responses appear to be the cause of AMS. However, the opposing mechanisms (insufficient versus overshooting/maladaptive) may account for inconsistent results regarding biomarkers and physiological/psychological correlates of AMS, highlighting the need to investigate the possibility that distinct AMS subtypes exist.

## 4 Putative biomarkers of acute mountain sickness

AMS can severely reduce the capacity to work and perform challenging mountain sports-related activities, predisposing for accidents. In addition, it can hamper the enjoyment of mountain activities and may be related to HACE development. Therefore, a better understanding of the disease, specific risk factors, the mechanisms involved and better approaches to treat or prevent AMS are needed. Since the diagnosis of AMS relies primarily on self-reported symptoms, the characterization of specific and more objective biomarkers is essential. Although much research on biomarkers in AMS has been conducted, few are generally accepted as specific for AMS and no biomarkers have been validated for routine use to diagnose AMS. In this section, we summarize recent developments in the research of AMS biomarkers as a basis to discuss how the field should develop to advance clinically useful applications and how to investigate potential AMS subtypes.

### 4.1 Search strategy

When discussing biological AMS biomarkers in the following section, we refer to quantifiable molecules providing information on pathological processes of AMS or allowing the prediction or definition/diagnosis of AMS. For the primary literature search on this class of biomarkers (the results are presented in Table 1, Table 2, Table 3), we did not include genetic markers (except for gene expression markers, i.e., RNAs) and we did not consider systemic physiological and psychological correlates of AMS but focused on molecules that quantitatively change in association with AMS. These molecular biomarkers for AMS have been investigated primarily in different body fluids, mainly in blood. To study AMS-related changes in the proteome, often enzyme-linked immunosorbent assay (ELISA), gel electrophoresis and mass spectrometry technologies have been used. To investigate the association between AMS and gene expression, RNA sequencing and qPCRs have been most commonly employed. In addition, the application of cytokine profiling approaches and other biochemical assays, as well as metabolomics contributed to shedding more light on the relation of AMS with levels or activities of specific molecules. However, despite a large body of literature on the topic, no biomarkers or sets of biomarkers have yet been successfully integrated in routines to diagnose AMS.

**Table 1:** Transcriptional changes (transcriptomics and qPCRs) associated with acute mountain sickness (AMS) in humans.

Tissue sample, method (Ref)	Study sample	Hypoxia/altitude exposure ascent information (AMS assessment)	Differentially expressed genes (DEGs) and potential applications as biomarkers
Blood, RNA sequencing [70]	113 Chinese lowlander participants (18-63 years), 9 women, 104 men; RNA sequencing was performed on 35 with and 13 without AMS.	During an exposure to 4104 m (no information on ascent reported, 23 days spent at this altitude), 56 developed AMS (LLS).	583 upregulated and 104 downregulated genes were found in those with AMS: 5 hub genes were identified as potential predictive biomarkers for AMS: *BCL2L1* (involved in cell survival)*, DCAF12* and *CDC34* (involved in targeted degradation)*, PINK1* (regulation of mitochondrial function and mitophagy)*,* and *UBB* (involved in proteostasis).
PBMCs, RNA sequencing [71]	Real altitude group (training cohort): N = 18 (23 ± 6 years), 5 women, 13 men.	In the real altitude study, participants were transported from 50 m to 2000 m by aircraft, where they received supplemental oxygen. The next day they ascended to 4300 m by car (in about 2 h). In the hypobaric chamber study, participants were brought to a simulated altitude of 4300 m within a 15-min period; at real altitude, 28% developed severe AMS (ESQ/AMS-C); in the hypobaric chamber 50% developed severe AMS.	A linear Support Vector Machine algorithm was used to identify putative diagnostic and predictive biomarker panels. *HBA1*, *HBA2*, and *HBB*, *PDE5A* regulation differentiated between AMS+ and AMS-. *TNNT1* was among the best diagnostic and predictive gene for AMS. CREB signaling in neurons, GPCR and phagosome formation pathways activated in AMS+.
	Hypobaric chamber group (validation cohort): 10 young healthy adults (23 ± 6 years), 2 women, 8 men. Ethnicities/country of origin not reported.		

Saliva, qRT-PCR [72]	124 male healthy Chinese lowlanders (20-23 years).	At a simulated altitude of 4500 m for 12 h, 75 (about 60%) developed AMS (LLS), 49 not. How rapidly participants were exposed to the final altitude is not reported.	Salivary miR-134-3p and miR-15b-5p levels at 200 m were higher in those, who had AMS at 4500 m, representing potential predictive biomarkers. Peripheral oxygen saturation and cerebral tissue oxygenation (assessed by NIRS) were lower in those who developed AMS.

Serum, qRT-PCR [73]	Transcriptomic analysis set: 10 healthy young (20-23 years) men. Protein validation set: 22 healthy young (22-32 years) men. Ethnicities/country of origin not reported.	Participants were rapidly transported from 1300 m to 5300 m by bus (during 72 h, 1 day was spent at 3000 m), 5 developed AMS (AMS+, using LLS), 5 not (AMS-). In the validation set 12 AMS+, 10 AMS-.	Potential associative biomarkers were explored: 1164 DEGs in AMS+ and 1322 DEGs in AMS-, with only 328 overlapping between AMS+ and AMS-.The anti-inflammatory cytokine IL-10 was lower in AMS+, pro-inflammatory cytokines IF17F and CCL8 were higher in AMS+.


Whole blood, plasma (qPCRs) [74]	109 healthy male lowlanders (17-35 years). 22 were randomly selected for microarrays (13 with AMS) and 87 for qPCR confirmations (41 with AMS). Ethnicities/country of origin not reported.	Participants were transported from 200 to 3648 m by train within 48 h, where they stayed for 5 days. Of the 87 participants selected for qPCR confirmations, about 47% had AMS (LLS).	31 differentially expressed microRNAs were identified as potential associative biomarkers in subjects with AMS. By multiple regression analysis, a set of microRNAs was found differentiating AMS from Non-AMS: miR-369-3p, miR-449b-3p, and miR-136-3p (92.7% sensitivity and 93.5% specificity). No differences between AMS and non-AMS for oxygen saturation and heart rate (except for a higher heart rate in AMS on day 3).


Notes: *AMS+: acute mountain sickness (AMS) susceptible individuals; ESQ: Environmental Symptoms Questionnaire;* HBA1*,* HBA2*, and* HBB*: Hemoglobin-related genes; GPCR: G-protein coupled receptor signaling pathways; LLS: Lake Louise Score; NIRS: near-infrared spectroscopy; PBMCs: peripheral blood mononuclear cells; PDE5A: phosphodiesterase 5A gene; qRT-PCR: quantitative reverse transcription polymerase chain reaction;* TNNT1*: troponin T1, slow skeletal type*.

**Table 2:** Proteomic/metabolomic changes associated with AMS in humans.

*Tissue sample, method (Ref)*	*Study sample*	*Hypoxia/altitude exposure, (AMS assessment)*	*Differentially expressed proteins/differential metabolite levels in AMS, and physiological observations, type of biomarkers*
Red blood cells from peripheral blood samples [75]	16 male participants (21−32 years) born and raised at altitudes below 2000 m in China.	Fast ascent to 4500 m (by train, in 48 h); 8 developed AMS (LLS), 8 remained asymptomatic.	Red blood cells of those with AMS had higher oxidative stress and disrupted membrane integrity; altered expression/phosphorylation of proteins related to energy metabolism (TCA) glycolysis), iron metabolism and the cytoskeleton was observed. Associative/pathophysiological biomarkers.
Plasma proteomics, metabolomics and other techniques [36]	83 male Chinese participants (median 23 years, IQR: 21–24).	Transported to 3650 m from 500 m by train in 34.5 h. Of 83 participants, 41 had no AMS (LLS), 42 had AMS (37 mild AMS and 5 moderate AMS); 66 samples were used for final analyses (36 non-AMS, 30 AMS).	Machine-learning analysis identified a multidimensional biomarker panel comprising the proteins ACSL4, IGKV1D-16, F13B, PSAP, PVR, and MMRN2 together with the metabolites 2-methyl-1,3-cyclohexadiene, calcitriol, 4-acetamido-2-amino-6-nitrotoluene, and 20-hydroxy-PGE2, which discriminated individuals who developed AMS from those who did not. Predictive biomarkers.
Plasma, proteomics [76]	158 male Chinese lowlander participants (median 22 years, IQR: 21-24), that had not traveled to high altitude within the previous 6 months; divided into discovery cohort (N = 40, age: median: 23 years, IQR: 22-26) and validation cohort (N = 118, median 22 years, IQR: 21-23).	Staged ascent by car (1400 m - 3700 m: 7-day acclimatization, then to 5000 m, within 10 h) with at 3700 m. At 3700 m: discovery cohort: 8 AMS (LLS), 32 non-AMS, validation cohort: 18 AMS, 100 non-AMS. At 5000 m: discovery cohort: 20 AMS, 20 non-AMS, validation cohort: 65 AMS, 53 non-AMS.	Pathways of granulocyte activation, neutrophil mediated immunity, humoral immune response and others differed depending on AMS. Higher levels of the proteins AAT, SAP and LTF in those who developed AMS already at 3700 m compared to no AMS; higher levels of HSP90-α and SAP in those who developed AMS at 5000 m only compared to no AMS, at low altitude. Predictive biomarkers.
Plasma, proteomics [77]	53 male Chinese lowlander participants (17-22 years).	Rapid ascent from ∼1000 m to ∼4000 m; 30 developed AMS (LLS).	Altered levels of enzymes related to energy metabolism pathways (nitrogen metabolism, pentose phosphate pathway, glycolysis, gluconeogenesis) in those with AMS, higher levels of proteins related to cytokine–cytokine receptor interaction and TNF signaling pathways, lower for bicarbonate transport. ADAM15-expression was suggested as protective, PHGDH-expression as predictive and TRAF2-expression as a diagnostic biomarker.
		Discovery cohort: 10 AMS individuals (20 paired samples). Validation cohort: 30 AMS and 23 non-AMS individuals (106 paired samples).	
Urine, untargeted NMR-based metabolomics [78]	17 healthy, unacclimatized, physically active male participants (18–42 years). Ethnicities/country of origin not reported.	Rapid ascent from sea level to 4300 m following air travel, supplemental oxygen was provided during transit; residence at altitude for 21 days; 11 developed moderate/severe AMS (LLS), 6 had no or mild AMS.	Metabolite levels, mainly related to energy metabolism, were significantly changed compared to sea-level on the first (creatine higher in AMS-) and 18th day (creatine and acetylcarnitine higher in AMS-, hypoxanthine and taurine higher in AMS+) at altitude. In addition, creatine, acetylcarnitine and 4-hydroxyphenylpyruvate levels were higher in AMS + at sea level compared to AMS-, while the reverse was true for taurine, N-methylhistidine and hypoxanthine, potentially representing predictive biomarkers.
Plasma, proteomics [79]	23 healthy Chinese participants (age range: 25-35 years) mainly living at 400 m or lower. Sex/gender not reported.	Rapid flight ascent (400 to 3800 m in approx. 3 h), followed by three 5-min submaximal exercise challenges 2 h after arrival. AMS (LLS) status in the full cohort not reported; proteomic analyses were performed on 14 participants selected from the highest (AMS+, n = 7) and lowest (AMS−, n = 7) LLS scorers.	Lower levels of proteins related to TCA cycle, glycolysis, ribosome, and proteasome pathways in AMS- at altitude present potential associative biomarkers.
Plasma: metabolomics, ELISA [80]	60 male Chinese participants (21.8 ± 1.8 years) not previously exposed to altitude.	Staged ascent by bus from 1400 m to 5300 m over 72 h, with a 1-day rest at 3000 m. For metabolome analysis, 10 participants with the highest (AMS+) compared with the 10 participants with the lowest LLS scores.	44 metabolite levels and four enzymes changed in subjects exposed to high altitudes for the first time; 5 metabolites (sphingomyelin, phosphatidylcholine, glutamic acid, glyceric acid, and 12,13-diHOME) were suggested to be predictive biomarkers for AMS, since their levels were higher at baseline in individuals, who developed AMS later. Trends of higher heart rates and lower oxygen saturation in AMS+ were not significant.
Plasma, proteomics [81]	29 healthy (mainly Caucasian/admixed Caucasian) participants (age not reported for the overall cohort). A subgroup of 20 was analyzed: 17 males, 3 females; those with AMS (N = 10, 27.8 ± 2.3 years) and without AMS (N = 10 (29.4 ± 2.4 years).	9-h simulated altitude corresponding to 1609 to 4875 m plus 4 x 30-min submaximal cycling bouts. The 20 individuals with the highest and lowest LLS scores after 9 h were analyzed.	AMS was associated with increased antioxidant proteins (e.g., peroxiredoxin 6, glutathione peroxidase, and sulfhydryl oxidase 1: associative biomarkers) whereas AMS-resistant individuals showed stronger acute inflammatory pathway activation. Associative/pathophysiological biomarkers.

Notes: *AAT: alpha-1-antitrypsin; ADAM15: ADAM metallopeptidase domain 15; AMS+: AMS susceptible individuals; AMS-: AMS-resistant individuals; CCL8: Chemokine (C–C motif) ligand 8; DEP: differentially expressed protein; ESQ: Environmental Symptoms Questionnaire; HSP90-α: heat shock protein HSP 90-alpha; LLS: Lake Louise Score; LTF: lactotransferrin; PHGDH: phosphoglycerate dehydrogenase; qPCR: quantitative reverse-transcription polymerase chain reaction; SAP: serum amyloid P-component; TCA: tricarboxylic acid; TRAF2: TNF receptor-associated factor 2*.

**Table 3:** Other biological biomarkers associated with AMS in humans.

*Tissue sample, method (Ref)*	*Study sample*	*Hypoxia/altitude exposure ascent information, (AMS assessment)*	Main biomarker findings; biomarker type
***Metabolic, endocrine and neuroendocrine biomarkers***			
ELISA of plasma specimens plus mouse/cell mechanistic experiments [82]	40 healthy male Chinese participants (18-25 years).	Transported from 200 m to 4260 m; hypobaric hypoxia/high-altitude exposure; 36 (90%) had AMS (LLS).	Pre-ascent plasma levels of the endothelial protective factor 5-methoxytryptophan (5-MTP) levels were negatively correlated with subsequent AMS severity. Predictive (susceptibility) biomarker.
Venous whole blood, plasma and serum; chemiluminescence immunoassay and ELISA [83] (AMS incidences reported in Ref. [84])	32 unacclimatized soldiers (23±4 years); 1 woman, 31 men. Ethnicities/country of origin not reported.	Ascent to 4300 m by hiking (active group, n = 16) or vehicle transport (passive group, n = 16) and staying for 4 days. AMS (ESQ- AMS-C) peaked on the first day: active group: 81% AMS, passive group: 69% AMS.	Blood glucose, insulin, epinephrine and norepinephrine were positively correlated with AMS-C scores. Associative/pathophysiological biomarkers.
Serum chemiluminescence immunoassay, automatic biochemical analyses and blood cell analyzer [37]	100 healthy euthyroid Chinese lowlanders; 70 were analyzed: 40 women, 30 men (median 29 years (IQR 25-39).	Rapid ascent by aircraft from Beijing (44 m) to Lhasa (3650 m) in approximately 5 h. Training set (80% of participants): AMS (LLS) n = 31, non-AMS n = 25. Residual cases used as validation set.	Higher baseline TT3 and FT3/FT4 ratio independently predicted AMS in multivariable logistic regression (OR 2.47 and 3.43, respectively). Predictive (susceptibility) biomarkers.
Arterial whole blood, venous plasma, blood gas analysis and HPLC [85]	18 lowlander participants (18-35 years); 8 women, 10 men. Ethnicities/country of origin not reported.	Normobaric hypoxia (12% O_2_; equivalent to 4600 m) for 8 h followed a baseline at 20% O_2_; how rapid the switch was, was not reported. AMS (LLS) n = 8; non-AMS n = 10.	Arterial epinephrine was higher at baseline and during hypoxia in participants who developed AMS; blood gases did not differ by AMS status. Potetial predictive/or associative/pathophysiological biomarker.
Plasma commercial assays, osmometer, creatinine clearance and radioimmunoassays [34]	Of 51 exposed participants; those with highest and lowest LLS-values were analyzed: AMS n = 16 (27±1 years), non-AMS n = 16 (28±1 years). Sex/gender, ethnicities/country of origin and total cohort age not reported.	Simulated altitude of 4880 m for 8-12 h (rapidity of switch to hypoxia NR). Comparison of the 16 highest LLS scorers (LLS 7.4, range 5-11) with the 16 lowest LLS scorers (LLS 1.0, range 0-2.5).	Plasma renin activity, aldosterone and ANP did not differ by AMS status. ADH correlated with AMS severity and fluid retention: ADH decreased in non-AMS and increased in AMS. Associative/pathophysiological biomarker.
Plasma commercial assays [86]	19 male mountaineers (38±12 years. Ethnicities/country of origin not reported.	Participants trekked to 5100 m over 20±5 days and remained there for 7±5 days. AMS n = 5; non-AMS n = 14.	Resting and exercise plasma cholecystokinin increased at altitude, with a stronger increase in participants with AMS. Associative/pathophysiological biomarker.
***Immune and inflammatory biomarkers***			
Whole blood flow cytometry [87]	205 male lowlanders (20±2 years). Ethnicities/country of origin not reported.	Rapid travel by aircraft from 200 m to 3600 m. Mild AMS (LLS) n = 71, severe AMS n = 17 and non-AMS n = 117 on day 1.	Severe AMS was associated with reduced frequencies of eosinophils, basophils, plasmacytoid dendritic cells and CD4^+^ T cells. Associative/pathophysiological biomarkers.
Plasma, Bio-Plex MAGPIX system [88]	48 male soldiers; sAMS median 24 years (IQR 22-31), mAMS median 23 years (IQR 21-26), non-AMS median 22 years (IQR 20-25). Ethnicities/nationalities not reported.	Staged ascent by car from 1400 m to 3700 m, 7 days at 3700 m, then ascent to 5000 m within 10 h sAMS: AMS (LLS) at 3700 m (n = 8); mAMS: no AMS at 3700 m but AMS at 5000 m (n = 17); non-AMS n = 23.	Low-altitude TNF-alpha predicted AMS at 3700 m; IL-2 and IL-17A predicted AMS after ascent to 5000 m. Predictive (susceptibility) biomarkers.
Plasma, ELISA [89]	34 participants (21-58 years); 19 women and 15 men. Ethnicities/nationalities not reported.	Transported by bus from 555 m to 3150 m within 3 h. AMS (LLS) n = 18; non-AMS n = 16.	E-selectin, CRP, MCP-1 and S100B increased and VEGF decreased at 3150 m, but E-selectin, CRP, MCP-1 and S100B were not associated with AMS. ANP and VCAM-1 were higher and PAI-1 lower in AMS at 3150 m; BNP was higher and VEGF and PAI-1 lower after descent. LLS was positively correlated with ANP and VCAM-1 levels, negatively with PAI-1 levels at 3150 m. Associative/pathophysiological biomarkers.
Serum cytokines, chemokines and growth factors by Luminex system [42]	15 healthy mountaineers (35±14 years); 6 women, 9 men. Ethnicities/country of origin not reported.	Participants trekked from 869 m to 5050 m over 14 days. AMS (LLS) n = 8; non-AMS n = 7.	Multiple cytokines, chemokines and growth factors changed with ascent. Serum VEGF was higher in AMS + at baseline and increased more with ascent than in AMS-. Predictive and associative biomarkers.
Plasma cytokine array [90]	23 Chinese lowlanders (n = 14 analyzed, 25-35 years); sex/gender not reported.	First study: rapid ascent to 3800 m in 3 h by aircraft. Second study: ascent to 4300 m by car over 3 days. Cytokine analysis compared in those with highest LLS (n = 7) and lowest LLS (n = 7) after 9 h at 3800 m.	75 differentially expressed molecules were identified; IGFBP-6, SAA1, Dkk4 and IL-17RA were reported as the most predictive for AMS. Predictive and diagnostic biomarkers.
Plasma, Bio-Plex system and ELISA [26]	29 participants living at about 1650 m; analyzed subgroups: AMS-susceptible n = 10 (28±2 years) and AMS-resistant n = 10 (29±2 years). Sex/gender, ethnicities/country of origin and total cohort age not reported.	Three 10-h hypobaric chamber exposures simulating 4875 m, separated by at least 3 weeks; trials used placebo, acetazolamide or dexamethasone. Analysis used the highest and lowest LLS scorers.	AMS-resistant participants showed stronger anti-inflammatory response patterns, including higher IL-1RA, HSP-70 and adrenomedullin; IL-10 was greater in AMS susceptible participants. Acetazolamide and dexamethasone both promoted IL-1RA and HSP-70 upregulation in AMS-susceptible individuals. Associative/pathophysiological biomarkers.
Plasma, immunoassay [91]	19 healthy volunteers (34±8 years); 5 women, 14 men. Ethnicities/country of origin not reported.	Exposure to 8 h of 10% normobaric oxygen after 4 days of either high-carbohydrate (68% CHO) or normal-carbohydrate (45% CHO) diet. AMS (LLCQ) group sizes NR.	Hypoxia and diet did not alter IL-1 beta, IL-6, IL-8 or TNF-alpha concentrations; no correlations with AMS symptoms were found. No biomarker association observed.
***Endothelial, vascular and cardiac biomarkers***			
Plasma, ELISA [92]	52 lowlanders (35±12 years), 17 women, 35 men. Ethnicities/country of origin not reported.	Transported by car to 2200 m for 2 days, then hiked to 3000 m; AMS (LLS) assessed 24, 48 and 72 h after arrival. AMS n = 13; non-AMS n = 39.	8 protein and peptide biomarker candidates were tested: MMP9 and substance P were significantly higher in people with AMS, while no significant differences were found for BNP, HIF-1α, NGAL, MMP-3, sestrin 2, and urotensin-2. No sex- and age-differences regarding AMS. No differences in blood pressure, heart rate and oxygen saturation. Diagnostic/associative biomarkers.
Plasma, ELISA [38]	12 subjects (22–56 years); 6 women, 6 men. Ethnicities/country of origin not reported.	Participants were brought to by car to 1400 m, then ascended to 3200 m and on the next day to 3700 m; AMS (LLS) n = 7 during first ascent. 6 participants received dexamethasone for a second ascent 48 h later.	Plasma VEGF increased at altitude in participants with AMS and decreased in those without AMS; oxygen saturation did not differ between groups. Associative/pathophysiological biomarker.
Plasma, ELISA [93]	261 male soldiers (21±2 years). Ethnicities/country of origin not reported.	Ascended by car from 600 m to 2600 m and the next day to 4300 m. Mild AMS (LLS) n = 54, severe AMS n = 22, non-AMS n = 185.	At 3500 m, plasma osteopontin levels significantly increased in soldiers without AMS, did not change in those with mild AMS and decreased in severe AMS. SOD decreased in soldiers with AMS, MDA was higher in severe AMS, compared to subjects with no AMS. Severe AMS was linked to higher heart rate and lower peripheral oxygen saturation. Associative/pathophysiological biomarkers.
Plasma, immunoassays [94]	48 trekkers (35±9 years). Ethnicities/country of origin and sex/gender not reported.	Participants were transported to 3600 m by aircraft, then performed a 10-days trek. Samples were taken after ascent at 3833, 4450 and 5129 m; 20 participants also provided sea-level samples. AMS (LLS) prevalence was 27.1% at 3833 m, 8.7% at 4450 m and 37.8% at 5129 m.	LLS correlated positively with IL-6 and endothelin-1 and negatively with oxygen saturation; IL-17A was not correlated with LLS. Associative/pathophysiological biomarkers.
Whole blood and serum, commercial assays [95]	48 trekkers (35±9 years). Ethnicities/country of origin and sex/gender not reported.	Participants were transported to 3600 m by aircraft, then performed a 10-days trek. Assesments post-trekking and at rest at 3833, 4450 and 5129 m. At 3833 m, AMS (LLS) n = 20 during exercise and n = 13 at rest; severe AMS n = 13 during exercise and n = 3 at rest.	BNP/NT-proBNP, cTNT and CRP increased with altitude, but only BNP and NT-proBNP were statistically associated with AMS. Associative/pathophysiological biomarkers.
Whole blood and serum, commercial assays [96]	20 trekkers; 36±2 years; 9 women, 11 men. Ethnicities/country of origin not reported.	Participants were transported to 2840 m by aircraft, then performed a 10-days trek. They ascended to a maximum of 5643 m; blood was assessed at 4270 m before and 5150 m after ascent. Severe AMS (LLS) at 5150 m n = 4; other group sizes NR.	BNP and NT-proBNP increased at altitude during rest and after exercise; BNP was higher in trekkers with severe AMS at 5150 m. Associative/pathophysiological biomarker.
***Neurological, blood-brain barrier and breathprint biomarkers***			
Plasma, single-molecule array (Simoa) [97]	63 healthy adults (25±4 years); 27 women, 36 men. Ethnicities/country of origin not reported.	Participants were exposed 2 times to simulated altitude (4500 m, F_I_O_2_ = 12,6%) for 12 h each, with or without preacclimatization (1h per day for 7 days before the 2nd exposure). 24 had AMS after the 2nd exposure.	pNfL increased more in AMS cases and was significantly higher in samples of people with AMS than in people without AMS, without preacclimatization effects.observed. Associative/pathophysiological biomarker.
Exhaled breath, hand-held vapor analyzer/electronic nose [98]	37 participants enrolled (18 Sherpas with confirmed Tibetan ancestry; median 25 years, 19 primarily European lowlanders with no Tibetan/Andean/Ethiopian ancestry and residing below 1300 m; median 31 years). While it is stated that women/men ratios were similar in both groups, precise sex/gender ratios not reported.	Participants were transported to 2800 m by aircraft, then performed a 19 days trek. At 3500 m, AMS (LLS) developed in 4 lowlanders (1 excluded for other reasons). At 5300 m, AMS n = 11 among the remaining 32.	Carbon-based volatile-organic compounds were assessed with a hand-held vapor analyzer from exhaled breath, generating a ‘breathprint’ (without identifying molecular components). Principal component analysis allowed classification of AMS and non-AMS at 5300 m with a sensitivity of 46% and a specificity of 81%. Diagnostic biomarker signature.
Plasma, chemiluminescence immunoassay [99]	12 trekkers (22-56 years); 6 women, 6 men. Ethnicities/country of origin not reported.	Participants were driven to 1400 m, and then trekked to 3700 m over 2 days. AMS (LLS) n = 7; non-AMS n = 5.	S100B increased with altitude but did not correlate with AMS. No AMS biomarker association observed, but a possible underestimation due to the small simple size is discussed.
Blood, commercial assays [100]	Of 52 trekkers (36±1 years), 22 women, 30 men. Ethnicities/country of origin not reported.	Trekking started at 3400 m, transport there not reported. During the 10-day trek a maximum altitude of 5150 m (for a subset of 20 the maximum was 5643 m). 46 reached the highest study point of 5150 m: severe AMS (LLS) n = 7, mild AMS n = 16, non-AMS n = 23.	CRP and NGAL increased at altitude; NGAL was higher with more severe AMS, whereas CRP was not significantly related to AMS. Potential associative/pathophysiological biomarker.
***Oxidative stress and redox biomarkers***			
Oxidative damage and antioxidant profiling in plasma, serum, erythrocyte and leukocyte samples [65], methods in [101]	10 male participants; (median 30 years, IQR 25-33). Ethnicities/country of origin not reported.	Participants ascended from 655 m to by vehicle to 3570 m and then to 4220 m on foot over 36 h. AMS (LLS) n = 10; non-AMS n = 0.	Post-expedition MDA increased and positively correlated with AMS severity; glutathione peroxidase decreased. Total antioxidant capacity, advanced oxidation protein products, methionine sulfoxide, catalase and SOD did not change. Associative/pathophysiological biomarkers.
EPR spectroscopy for serum lipid-derived free radicals, ozone-based chemiluminescence for nitrative stress and fluorometry/HPLC for plasma antioxidants [66]	11 healthy men (27±4 years). Ethnicities/country of origin not reported.	Normobaric hypoxia (12.9% O2; corresponding to 3800 m) for 9 h (rapidity of switch NR). Mean LLS increased from 0±0 in normoxia to 3±2 in hypoxia; AMS was also assessed using the ESQ-AMS-C.	S100B increased and NSE decreased during hypoxia; S100B changes correlated with AMS severity. AMS/headache scores correlated with 3-nitrotyrosine and other oxidative/nitrosative stress markers. No correlation between hemodynamic (including global cerebral bloodflow) or oxidative metabolic indexes and AMS scores. Associative/pathophysiological biomarkers.
Exhaled breath condensate and serum liquid chromatography and spectrophotometry [102]	10 male soldiers (29±4.3 years). Ethnicities/country of origin not reported.	Soldiers climbed to 6125 m during a 15-day expedition; AMS (LLS) assessed at 5000 m: AMS n = 8; non-AMS n = 2.	MDA and H_2_O_2_ increased at higher altitude; MDA was positively correlated with AMS scores. Associative/pathophysiological biomarkers.
Serum/plasma, mass spectrometry and commercial assays [103]	49 participants; biomarker subset n = 24 (AMS n = 14). Ethnicities/nationalities and sex/gender not reported.	Rapid passive ascent from sea level: to 3200 m, then active ascent to 3611 m, one night there, then active ascent to 4559 m within 40 h. AMS (LLS) n = 25; non-AMS n = 24.	Total creatine phosphokinase increased specifically in those with AMS. F2-isoprostanes, NSE and tested proinflammatory cytokines did not differ by AMS status. Associative/pathophysiological biomarkers.

Notes: *ACSL4: Acyl-CoA synthetase long-chain family member 4; AMS+: acute mountain sickness (AMS) susceptible individuals; AMS-: AMS-resistant individuals; AMS-C: AMS-Cerebral score (obtained from the shortened version of the Environmental Symptoms Questionnaire (ESQ)); ANP: atrial natriuretic peptide; BDNF: brain-derived neurotrophic factor; BNP: brain natriuretic peptide; CRP: c-reactive protein; cTNT: cardiac troponin T; ELISA: enzyme-linked immunosorbent assay; EPR: Electronic magnetic resonance; ERS: Electron paramagnetic resonance; ESQ: Environmental Symptoms Questionnaire; F13B: coagulation factor XIII B subunit; FEV1: forced expiratory volume in 1 s; FVC: forced vital capacity; H2O2: hydrogen peroxide; HIF-1α: hypoxia-inducible factor-1 alpha; HSP-70: heat shock protein 70; IGKV1D-16: immunoglobulin kappa variable 1D-16; IHT: intermittent hypoxia training; IL-10: Interleukin-10; IL-*1RA*: Interleukin-1receptor agonist (sic); IQR: interquartile range; LLS: Lake Louise Score; LLCQ: Lake Louise Consensus Questionnaire* [104]*; MCP-1: monocyte chemotactic (or chemoattractant) protein-1; MDA: Malondialdehyde; MIP-1β, macrophage inflammatory protein-1β; MMP-3: matrix metalloproteinase-3; MMP-9: matrix metalloproteinase-9; MMRN2: multimerin-2; MRI: magnetic resonance imaging; NGAL: neutrophil gelatinase-associated lipocalin; NMR: nuclear magnetic resonance; NR: not reported; NSE: neuron-specific enolase; PAI-1: plasminogen activator inhibitor-1; pNfL: Plasma neurofilament light chain; PSAP: prosaposin; PVR: poliovirus receptor; S100B: S100 calcium-binding protein B; SOD: superoxide dismutase; TNF-α: tumor necrosis factor alpha; VCAM-1: vascular cell adhesion molecule-1; VEGF: vascular endothelial growth factor*.

Aiming to provide an extensive overview on biomarkers investigated for their potential use to predict or diagnose AMS and suitable to speculate on potential AMS subtypes, we searched PubMed for publications linking “acute mountain sickness” with “biomarkers” until 16th of March 2026. Inclusion criteria were reported biological/molecular biomarkers from people with AMS. Exclusion criteria were a lack of precise altitude or population characterization (e.g., some database studies) or studies investigating genomic risk factors for AMS (e.g., GWAS). Studies focusing solely on animal experiments were not considered (although many transcriptomic and proteomic analyses demonstrated changes in animals exposed to hypoxia in different tissues, including the brain, due to the poor mechanistic understanding and the lack of established biomarkers, it is difficult to link such changes to human AMS). In addition, studies investigating AMS only before 3 h after altitude exposure were not included. Moreover, we searched Google scholar and used backward reference searches of the literature on AMS and forward citation tracking. For the exploratory literature searches, we also included search terms like transcriptomics, proteomics, metabolomics and cytokines to not miss studies identifying AMS-related molecules without labeling them biomarkers.

### 4.2 Transcriptional, proteome and metabolome characteristics of acute mountain sickness

The advent of powerful OMICs and computational methods has allowed the molecular characterization of diseases in unprecedented detail. These techniques have also been used in some studies on AMS. A recent study investigated transcriptional patterns (by RNA sequencing) associated with AMS in peripheral blood mononuclear cells (PBMCs) in order to predict AMS susceptibility at low altitude [71]. AMS was assessed after exposure to an altitude of 4300 m for 12 h. Hemoglobin-related genes (*HBA1*, *HBA2*, and *HBB*) and phosphodiesterase 5A (*PDE5A*) were identified as potential predictors for AMS vulnerability. Components of the cAMP response element-binding protein (CREB) pathway exhibited differential regulation at low and high altitude depending on AMS. While CREB signaling was weaker in AMS-vulnerable people at low altitude, it became upregulated in comparison to non-vulnerable people at high altitude [71]. Another transcriptomic study identified genes related to energy metabolism (*PINK1)*, cell survival (*BCL2L1*) and homeostasis/degradation (*DCAF12, CDC34* and *UBB*) to be differentially regulated in individuals developing AMS compared to those without AMS, thus representing potential biomarkers [70].

Earlier studies mostly relied on quantitative polymerase chain reactions (qPCRs) to investigate the inter-relation of AMS and gene expression, as summarized in Table 1. Liu and colleagues, for example, found highly divergent expression patterns in subjects susceptible or resistant to AMS [73]. They observed that genes encoding anti-inflammatory cytokines, like interleukin 10 (IL-10), tended to be expressed less in AMS-susceptible individuals, while the opposite was true for pro-inflammatory cytokines. Some studies used computational approaches to identify AMS biomarkers from existing OMICs datasets [105,106]. One study used machine learning to identify a set of genes (involved in immune functions, extracellular matrix remodeling and cellular signaling) as optimal biomarkers from available RNA-sequencing data [105]. Another study [106] suggests the gene *STC1* to be differentially expressed (upregulated) in those developing AMS, based on the dataset described by Liu and colleagues [74] among others. *STC1* encodes the immune-cell regulating protein Stanniocalcin 1, which is involved in the regulation of calcium and phosphate homeostasis. Such studies are, however, frequently difficult to interpret, as for example the selection of samples is not always clearly described, the secondary analyses may rely on small and heterogeneous samples and sometimes conditions such as altitude exposure or information in the AMS diagnosis are lacking.

Several studies investigated the differential expression of microRNAs in AMS [72,74]. MicroRNAs are noncoding, single-stranded RNAs of a length of about 22 nucleotides. They play important roles in cellular processes, regulating gene expression post-transcriptionally by binding messenger RNA. In one study, saliva samples taken before exposure to a simulated altitude corresponding to about 4500 m were analyzed [72]. The levels of the microRNAs miR-134-3p and miR-15b-5p, were found to be higher in those who later developed AMS in the simulated altitude [72]. In another study [74], 31 microRNAs that were differentially expressed in AMS were described. A specific set of them had good sensitivity/specificity values (Table 1), suggesting microRNAs to have a good potential as a clinically valuable biomarker for AMS.

Studies investigating differences related to AMS at the level of the proteome or metabolome are described in Table 2. Investigations of specific target molecules or other sets of biomarkers are summarized in Table 3.

### 4.3 Energy metabolism, oxidative stress and inflammatory biomarkers

Oxidative stress likely plays a role in high-altitude illnesses, including AMS [107]. This is suggested by increased levels of reactive oxygen and nitrogen species and oxidatively modified molecules, such as for example malondialdehyde (MDA) in breath condensate [102] or plasma [93] of mountaineers with AMS and by correlations of blood MDA levels and other markers of oxidative/nitrosative stress with AMS severity [65,66]. It is further supported by reports (randomized, double-blind, placebo-controlled trial, 18 subjects) that prophylactically ingested antioxidant vitamins (250 mg of l-ascorbic acid, 100 IU of dl-a-tocopherol acetate and 150 mg of alpha-lipoic acid, 4x per day) attenuated AMS severity at about 5200 m [108]. However, a larger (83 subjects) double blind, randomized, placebo-controlled study did not find a preventive effect of antioxidant supplementation (daily dose of 1 g l-ascorbic acid, 400 IU of α-tocopherol acetate and 600 mg of α-lipoic acid, divided in 4 doses) on AMS at 5200 m [109]. In a study on Chinese soldiers at 3500 m superoxide dismutase levels were lower in those with AMS [93], suggesting upregulation of antioxidant defense systems as a mechanism to counteract AMS in resistant individuals. However, this regulation may be transient, because although the levels of the antioxidant enzyme glutathione peroxidase were decreased in mountaineers developing AMS at 4220 m, no changes in superoxide dismutase levels and total antioxidant capacity were observed 24 h after altitude exposure [65]. Interestingly, plasma proteins related to antioxidant defense mechanisms were found to be upregulated in people with AMS after a 9-h exposure to hypobaric hypoxia of 4875 m, but not in those without AMS, which may indicate an overcompensation with negative outcome [81]. In contrast, anti-inflammatory factors, e.g., interleukin 1 receptor antagonist and heat shock protein-70 were higher in people resistant to AMS in the same conditions [26]. Medication like acetazolamide and dexamethasone appeared to support similar protein-regulation in AMS-susceptible individuals in this study [26]. These results suggest that insufficient anti-inflammatory responses may facilitate AMS. Inflammatory and immune responses are indeed supported by a big body of literature to be involved in AMS pathogenesis [73,76,77]. Inflammatory pathways are thought to be activated as a coping mechanism in hypoxia [36]. Accordingly, earlier studies reported an upregulation of individual pro-inflammatory cytokines like IL-6 to be associated with AMS [94], while other studies did not find such correlations for cytokines like IL-1 beta, IL-6, IL-8 and TNF-alpha [91,103]. Several cytokines have also been suggested as predictors for AMS, e.g., IL-2 and IL-17A, in newer studies [73,88]. The assessment of individual cytokines may, however, not be particularly useful for AMS prediction, since their expression in peripheral blood is frequently transient and low at baseline, and may be difficult to measure at a sufficient temporal resolution to correlate timely and reliably with AMS. In another study [87], immune cell profiles were associated with severe AMS on the first day at 3600 m: lower eosinophils, CD4^+^ T-cells, plasmacytoid dendritic cells and basophils and higher neutrophils were characteristic for severe AMS.

Recently, proteomics studies confirmed the involvement of inflammation in AMS. Guo and colleague conducted a study in 40 subjects, in which baseline LLS and fasting blood samples were obtained at an altitude of 1400 m, after which the participants traveled to 3700 m and 7 days later to 5000 m, both times by car [76]. Fasting blood samples and LLS scores were collected 36-48 h after arrival of the respective altitude levels. Based on their LLS scores, the subjects were retrospectively classified as either severe AMS-susceptible (developing AMS at 3700 m, 20%), moderately AMS-susceptible (developing AMS only at 5000 m, 50%) or without AMS. Inflammatory and immune related pathways were identified as differentiating best between subjects with or without AMS. Specifically, the expression of the proteins heat shock protein HSP 90-alpha (HSP90-α), serum amyloid P-component (SAP), alpha-1-antitrypsin (AAT) and lactotransferrin (LTF) were validated by ELISA and suggested as potential biomarkers at 1400 m and 3700 m. AAT, SAP and LTF were higher in severely AMS-susceptible individuals, HSP90-α and SAP were higher in moderately AMS-susceptible individuals already at 1400 m as compared to those not experiencing AMS at either altitude. These authors further identified proteins related to granulocyte activation, neutrophil mediated immunity and the humoral immune response to be most divergent regarding AMS [76], supporting inflammatory processes to be importantly implicated in AMS pathogenesis.

Yang and colleagues found clear changes in protein regulation in relation to the development of AMS at 4000 m and confirmed the involvement of inflammation (higher levels of proteins related to cytokine–cytokine receptor interaction and TNF signaling pathways in the plasma of individuals with AMS) [77]. In addition, they revealed energy metabolism pathways to be differentially regulated in those with AMS. Specifically, the regulation of HIF-modulator matrilin 3 (MATN3) and the cytoskeleton-regulation-associated protein myocilin (MYOC), as well as S100 calcium binding protein A12 (S100A12), involved in the regulation of inflammatory and immune responses, and ret proto-oncogene (RET) were found to be linked to the pathogenesis of AMS [77]. As biomarkers for the potential prediction of AMS at low altitude, these authors identified phosphoenolpyruvate carboxykinase 1 (PCK1), phosphoglycerate dehydrogenase (PHGDH), ribokinase (RBKS), S100A12, solute carrier family 4 member 1 (SLC4A1), and secreted protein acidic and cysteine rich (SPARC), which may reflect the adaptive capacity of the metabolism to adapt to altitude (reduced bicarbonate excretion for pH regulation in the plasma of subjects with AMS). Yang et al. suggested several proteins to represent potential diagnostic markers, including TNF receptor-associated factor 2 (TRAF2), ADP ribosylation factor 6 (ARF6), Epstein-Barr virus induced 3 (EBI3), GC vitamin D binding protein (GC), immunoglobulin superfamily containing leucine rich repeat 2 (ISLR2), MYOC, neuropilin 2 (NRP2), RBKS, RET and WAP, follistatin/kazal, immunoglobulin, kunitz and netrin domain containing 1 (WFIKKN1). These authors used machine-learning models to distinguish with high accuracy blood samples of individuals at low altitude who would or would not develop AMS and of individuals who were susceptible or resistant to AMS at high altitude. Those results confirm the potential of inflammation-related molecules and highlight the importance of molecules linked to energy metabolism as biomarkers for AMS.

Lu et al. also found differences in proteins of energy metabolism pathways in human plasma after 6-12 h of exposure to 3800 m^90^: in those resistant to AMS lower plasma levels of proteins of the tricarboxylic acid cycle but also of glycolysis were observed, which was not the case in those susceptible to AMS. In addition, reductions in proteins related to energy-intensive processes such as related to the ribosome or the cellular degradation system of the proteasome were observed [79]. This may suggest that in individuals resistant to AMS oxygen-intensive (e.g., tricarboxylic acid cycle) or energy-intensive processes are more effectively regulated by reducing energy demand in hypoxic conditions than in vulnerable individuals or that no excessive activation of HIF-pathways occurs [79]. Interestingly, another study suggests that creatine phosphokinase is specifically upregulated in those with AMS [103], possibly as a compensatory mechanism for energetic stress. A confirmation of the dynamic regulation of enzymes related to energy metabolism (however, rather an upregulation of glycolysis) and antioxidant mechanisms has been provided in rat cortex and hippocampus, acutely exposed to different doses of hypobaric hypoxia [110]. Metabolomics further suggest higher low-altitude creatine and acetylcarnitine levels, and lower hypoxanthine and taurine levels in urine samples of subject suffering from AMS compared to AMS resistant individuals, before ascending to an altitude of 4300 m [78], confirming changes in energy metabolism in dependence of AMS.

Li and colleagues [75] also found proteomic differences especially related to energy and iron metabolism in healthy men with AMS compared to those without AMS when analyzing the red blood cell proteome. Moreover, Langan and colleagues [83] showed altitude-related impairments of fasting glucose and insulin sensitivity to be positively correlated with AMS severity, probably contributing to cellular energy metabolism deficits in AMS.

In conclusion, components of redox regulation and oxidative defense, immune system activation and inflammatory responses and energy metabolism are among the most frequently identified targets of cellular hypoxia responses and have frequently been found to be differentially regulated in AMS-susceptible and AMS-resistant individuals, although the results regarding individual molecules are inconsistent. Overall, excessive oxidative stress responses (surprisingly including the upregulation of antioxidant enzymes – this may reflect a potentially insufficient or unsuccessful mechanisms counteracting oxidative damage) has frequently been linked to the development of AMS. Anti-inflammatory mechanisms counteracting immune responses and inflammatory processes related to high-altitude exposure appear to protect from AMS. In addition, the down-regulation of oxygen-dependent and energy-intensive processes could also protect from AMS by reducing energy deficits and the need for adaptations to compensate for reduced oxygen availability to fuel aerobic energy metabolism pathways. Taken together it appears plausible, that both too mild and too strong cellular responses promote unfavorable outcomes that may be associated with AMS development. Thus, insufficient hypoxic induction of immune/inflammatory responses may drive AMS in some individuals, while excessive upregulation – including of antioxidant defense systems – may contribute to AMS development as well.

### 4.4 Lipid and amino acid metabolism

In the urine of individuals at sea level, Sibomana and colleagues observed 4-hydroxyphenylpyruvate levels to be higher in people who did not develop AMS later when exposed to high altitude, while N-methylhistidine was higher in people that did develop AMS [78], suggesting differential changes in amino acid metabolism to be related to AMS pathogenesis. Using plasma metabolite profiling, Liao and colleagues found 44 metabolites to be significantly upregulated in unacclimatized men, when transported to 5300 m [80]. Among them were lipids and amino acids that were differentially higher in AMS-susceptible people before ascending to high altitude. One of them was the fat-derived (from linoleic acid) signaling molecule 12,13-diHOME, which has been described to have toxic and pro-oxidative properties and perturbs mitochondrial and neutrophil function [80]. The authors of this study found a general upregulation of free fatty acids, such as linoleic acid, arachidonic acid, eicosapentaenoic acid, docosahexaenoic acid, oleic acid, and palmitic acid, at high altitude, which could modulate immune and inflammatory processes, possibly contributing to AMS. Other potentially predictive metabolites for AMS (higher in AMS -susceptible individual at baseline) identified in this study were phosphatidylcholine, sphingomyelin, glutamic acid and glyceric acid [80].

In summary, changes in oxygen availability instantly affect cellular metabolism and these changes may be involved in AMS development. Amino acid and lipid metabolism are strongly inter-related with energy metabolism, redox-regulation and inflammatory responses discussed in the previous section. Therefore, alterations of related molecules are promising to help in the characterization of potential AMS subtypes and provide diagnostic and predictive information on AMS.

### 4.5 Other circulating factors; hormones, chemokines, growth factors and renal/cardiac damage

Many circulating factors have been identified as potentially differentiating between individuals at risk or to contribute to AMS diagnosis. Of particular interest are molecules with long-range signaling functions and damage markers of specific organs, which may provide information of specific organ vulnerabilities contributing to AMS development.

A recent study demonstrated a strongly increased AMS risk of higher thyroid markers in euthyroid adults [37]. When the subjects were rapidly transferred to 3650 m, those with high baseline levels of triiodothyronine and a high free triiodothyronine/free thyroxine ratio had a 2.5 or 3.4 odds ratio to develop AMS. These results suggest thyroid signaling as an important factor in AMS. Another tissue possibly involved in AMS-related signaling is bone tissue. The bone-derived cytokine osteopontin pays a role in a variety of biological processes, including bone-growth and immune function. Its plasma levels have been shown to rise with increasing altitude in healthy individuals, whereas no change or even a decline was observed in altitude sojourners that developed AMS [93]. This divergent, AMS-dependent regulation makes osteopontin an interesting biomarker for AMS. It may also suggest an impaired physiological regulation of this protein to be involved in AMS etiology.

During a trek to 5050 m, a dynamic regulation of various cytokines, chemokines and growth factors was observed [42], indicating possible inter-organ crosstalk involved in AMS. Interestingly, serum levels of VEGF, a protein importantly regulated by HIFs and a possibly important player in AMS, not only was higher in AMS-susceptible individuals before altitude exposure, but it also increased more during the trek, possibly indicating an overshooting HIF-response to be involved in AMS pathogenesis [42]. In another small study, Winter and colleagues observed increasing plasma protein levels of the HIF-regulated VEGF in individuals who developed AMS at 3700 m, while this was not the case in subjects resistant to AMS [38]. This observation may support an excessive hypoxia response to be implicated in AMS. Not all authors, however, did find associations between VEGF and AMS (e.g., Ref. [26]).

Neutrophil gelatinase-associated lipocalin (NGAL, a biomarker for kidney injury) is upregulated in conditions of oxidative stress and inflammation and therefore has been selected to be tested as a potential biomarker for AMS, alongside with the commonly used hepatic inflammation marker c-reactive protein [100]. The authors observed an upregulation of NGAL with increasing altitude in exposed individuals, with significant differences between those who developed AMS and those who did not at the highest observed altitude of 5150 m [100]. Although no such relationship was observed for c-reactive protein in this study, NGAL correlations with AMS further support the link of inflammation and AMS. It also may indicative of a role of kidney injury in some cases of AMS development, which could be related to dehydration, as another risk factor of AMS.

In addition, heart injury biomarkers have been implicated in AMS. BNP (brain natriuretic peptide) and NT-proBNP (N-terminal pro-B-type natriuretic peptide) are established biomarkers for heart failure. Mellor and colleagues tested, if their levels are also affected by AMS in 48 trekkers ascending a maximum altitude of 5129 m [95]. Like cardiac troponin T and c-reactive protein, BNP and NT-proBNP-levels in blood increased with increasing altitude. However, only BNP and NT-proBNP-levels significantly correlated with AMS, increasing more in those with AMS [95]. Similarly, associations of BNP and NT-proBNP with AMS severity were observed by Woods and colleagues [96]. A recent study also suggested lower levels of an endothelial protective factor (5-Methoxytryptophan) as predictive for AMS [82].

Overall, signaling molecules derived from various tissues may provide insights in the development of AMS and could inform about individual risk factors, derived from (pre-)pathological organ-deficiencies. The markers of specific organ damage (e.g., of the heart [96] and kidney [100]) that have been reported to be associated with AMS, raise the possibility that specific vulnerabilities of organs (e.g., kidney injury due to dehydration or exertion-related strain of the cardiovascular system) may promote differential developments of AMS progression or be causally involved in AMS development. Therefore, there may be AMS subtypes driven by, for example, kidney injury or cardiac stress, which may have differential progressions and may be characterized by differential biomarkers. This could explain discrepancies between studies. Future studies should attempt to link those molecules to specific at-risk populations and/or identify organs specifically vulnerable to hypoxia.

The findings of strong expression of HIF-regulated proteins like VEGF linked to AMS further support the possibility that excessive hypoxia responses are involved in AMS development.

### 4.6 Neurotransmitters and precursors, neuronal/brain damage markers

An organ principally involved in AMS clearly is the brain, and headache is widely regarded as the principal and a compulsory symptom of AMS [43]. The brain is particularly vulnerable to hypoxia [111] and the synthesis of key neurotransmitters depends on oxygen as well [112]. Accordingly, the regulation of several neurotransmitters/neuromodulators and their precursors at altitude has been implicated in the pathogenesis of AMS.

For example, during a hike to 3000 m, out of 8 selected protein and peptide biomarkers, the neuromodulator substance P and the extracellular matrix component MMP9 were identified as potential predictors for AMS [92]. The other tested molecules that were not associated with AMS in this study notably included HIF-1α and NGAL, highlighting the inconsistency of found associations of these molecules with AMS. Also, the neurotransmitter precursor tryptophan, as well as the neurotransmitter serotonin (involved in the regulation of mood and activity states), were shown to be increased in AMS and independent predictors for AMS with a promising specificity and sensitivity for diagnostic purposes [113]. Interestingly, tryptophan supplementation has been shown to improve hypoxic-stress-induced gastrointestinal problems in mice [114], indicating that tryptophan deficits may also be involved in gastrointestinal symptoms of AMS in some individuals. In contrast, the higher tryptophan and serotonin levels in blood of people with AMS [113] may indicate reduced activity of the oxygen-dependent kynurenine pathway, which would be linked to increased oxidative stress and energy metabolism deficits, if kynurenine levels are reduced as a consequence [115]. Another study [86] found a link of cholecystokinin, a neurotransmitter involved in the regulation of appetite- and digestion but also of anxiety, with AMS. While plasma cholecystokinin levels increased in general during a trek with a maximum altitude of 5100 m, they increased even more in those with AMS. Epinephrine levels in arterial blood were higher both at baseline and during simulated hypoxia exposure (4600 m) in individuals susceptible for AMS [85], reflecting another possible link between psychological stress/mood/anxiety and AMS [116].

Aside from neurotransmitters, brain-damage markers have emerged as potential AMS biomarkers. Plasma levels of the neuroaxonal damage marker neurofilament light chain, for example, were found to be higher in individuals with AMS as compared to controls [97]. This effect appeared to be independent of pre-acclimatization. Blood-brain barrier dysfunction has been discussed to contribute to the pathogenesis of AMS, although no clear evidence of an association of a disrupted blood-brain barrier with AMS is available for humans [1,117]. In a small sample (12 people), the blood-brain barrier dysfunction biomarker S100B increased at altitude, however, this increase was not significantly correlated with AMS [99]. While several cerebral damage markers and also oxidative stress may represent promising biomarkers for AMS, free radical-mediated neuronal damage measured from the peripheral circulation appears not to be a good biomarker for AMS [103].

Taken together, the exquisite vulnerability of the brain necessitates quick responses to hypoxia and both hypoxia itself as well as the hypoxia responses are associated with signaling alterations. Although these changes likely are involved in AMS, it is not well understood yet, how. We propose that hypoxia requires a well-calibrated hypoxia response for the brain to maintain its functional capacities and not suffer structural damage. If the metabolic challenges of ambient hypoxia remain unmet in the brain (insufficient hypoxia responses) this can rapidly lead to energetic deficit, oxidative stress and altered neurotransmission, all factors that are thought to be part of the conditions leading to AMS. Conversely, overshooting hypoxia responses, like excessively increased cerebral blood flow, VEGF overexpression (potentially leading to cerebrovascular and blood-brain barrier deficits) or maybe even excessive anti-oxidant mechanisms could be involved in AMS development.

### 4.7 Non-invasive biomarkers

Obtaining biomarkers non-invasively, e.g., from body fluids like saliva and urine, as well as breath analysis, is especially suited for the diagnosis of AMS or monitoring altitude-induced physiological and pathophysiological changes, especially during field studies or when diagnosing AMS in, e.g., mountaineering subjects, expeditioners, pilgrims, etc. Alterations upon exposure to altitude have been reported in non-invasively obtained samples, including changes in the salivary proteome after exposure to about 4200 m [118]: however, AMS was not reported in this study. Studies have also linked AMS to salivary [72] or urine [78] biomarkers, or to breath analyses [98,119]. Regarding breath analysis, Lacey and colleagues used a hand-held vapor analyzer, termed “electronic nose” to assess volatile-organic compounds from exhaled breath [98]. They demonstrated that a principal component analysis-based classification using the obtained unspecific (no molecular components are identified) “breathprints” is possible. However, the sensitivity of the method, when comparing 11 people with and 21 people without AMS after exposure to 5300 m amounted to only 46%. Another novel non-invasive potential biomarker for AMS is a changed retinal microvasculature, as indicated by higher retinal radial peripapillary capillary flow density in individuals with AMS, measured by optical coherence tomography angiography (OCTA) [120].

The identification of non- or minimally invasive sets of biomarkers is an urgent need to increase the diagnostic accuracy for practical applications in mountain environments and thereby facilitate treatment. The combination of such biomarkers with physiological and psychological correlates of AMS appears an especially important aspect for improving diagnostic accuracy.

## 5 Physiological and psychological correlates of AMS

AMS appears to be correlated with changes in vital/physiological parameters, such as ventilation and arterial oxygen saturation, autonomic nervous function, cerebral blood flow, increased brain volume, with psychological indicators, including psychological stress, fatigue or state/trait anxiety, and with body composition (e.g., body water [121] and body mass index [8]), although these differences are not observed in all studies. For example, increasing LLS and in particular fatigue as an isolated factor, have been shown to be associated with decreasing fat-free mass, total body water, and extracellular water [121]. Other potential physiological correlates of AMS – that are still not completely understood – include highly multifactorial cardiovascular parameters (such as heart rate and heart rate variability, blood pressure) or periodic breathing [122].

While these physiological correlates are important aspects to consider when developing biomarker sets to predict or diagnose AMS or to define subgroups of AMS, they are not entirely deterministic. Thus, although several physiological parameters have been linked to AMS, they are not of sufficient sensitivity and specificity to serve as reliable diagnostic markers by themselves. This is even true for arterial oxygen saturation. A slightly lower arterial oxygen saturation and arterial oxygen tension (hypoxemia) and mildly greater alveolar–arterial oxygen tension differences have commonly been observed on average in people with AMS as compared to those without AMS [1]. Hypoxia exposure is associated with cerebral blood flow changes [123], which is related to hypoxemia. Increasing hypoxemia in AMS has been shown to be associated with even greater enhancement of cerebral blood flow [124]. Although this seems to indicate that increased cerebral blood flow contributes to AMS pathogenesis, this has not yet been conclusively demonstrated. However, a study found increased cerebral blood flow through and diameter of the internal carotid artery and vertebral artery to be positively correlated with AMS in healthy adults exposed to a simulated altitude of 5000 m [125].

A recent study provided promising results for echocardiographic biomarkers of AMS, indicating especially increasing pulmonary artery diameter as a robust predictor of AMS [126]. However, the authors also reported pressure load, remodeling and systolic function of the right ventricle as altitude-dependent AMS predictors [126], suggesting those parameters to represent potentially useful features for the differentiation of AMS subtypes.

Periodic breathing and hypoxemia are closely associated and this has been shown especially in sleep studies [127]. Yet, ventilation variability in hypoxia, either awake or sleeping, is scarcely explored [128]. In addition, combining heart rate variability and ventilation variability may allow to assess respiratory sinus arrythmia. These three criteria are all closely linked to the control of ventilation which has been previously linked to AMS development. Future research should focus on determining their potential connection with AMS development.

Alterations of water homeostasis, dehydration and/or water retention, are other factors probably contributing to the development of AMS [129]. Hypoxia results in increased water and sodium secretion [129] potentially leading to dehydration, which has been reported to be associated with AMS severity [130]. Moreover, (early) water retention was found to favor AMS development [[32], [33], [34]], although conflicting data have also been reported [35]. Water retention has been linked to hypoxia-induced changes in circulating arginine vasopressin, aldosterone and/or atrial natriuretic peptide levels [34]. At high altitude, sympathetic activation can further alter renal regulation [131]. Furthermore, an involvement of the hypothalamic–pituitary–adrenal (HPA) axis and psychological stress response on water regulation and AMS development has been discussed [32,132,133]. However, a direct causal link between increased fluid intake, water retention, and AMS remains speculative; nevertheless, elevated extracellular water, together with factors such as altered endothelial permeability or upregulation of inducible NO synthase, may affect the blood-brain barrier, increase brain tissue volume, raise intracranial pressure, and thereby contribute to severe AMS symptoms [134]. In addition, fluid retention could limit renal bicarbonate excretion, which is necessary for the correction of hypoxia-induced respiratory alkalosis [135].

An MRI study found a slightly increased brain volume in subjects exposed to a simulated altitude of 4500 m (F_I_O_2_ = 0.12, 50% developed AMS), however, independent of AMS [136]. Increased intracerebral pressure or brain swelling may activate pain receptors in blood vessels or meninges around the brain. It is still debated if the mild brain swelling reported for AMS is sufficient to mechanically stimulate pain receptors, but it could trigger the release of nociceptive molecules, thereby contributing to AMS-associated headache [1].

A small hypoxic ventilatory response and a stronger desaturation, both in particular during exercise in hypoxia, have also been identified as independent predictors of severe high-altitude illnesses (including severe AMS and the much rarer HAPE and HACE) in a population of 1326 individuals sojourning at >4000 m [6]. The reported low levels of proteins of bicarbonate transport in subjects with AMS [77] might indicate a lower ventilatory response and thereby less pronounced respiratory alkalosis and less reason for compensatory bicarbonate secretion. Alternatively, it could also mean less efficient translational responses to hypoxia. This is supported by the findings of Barclay and colleagues, who identified rapid alkalinization and insufficient metabolic compensation by renal bicarbonate secretion as predictors of AMS [125]. Moreover, upper respiratory symptoms increase with altitude and are a potential risk factor for AMS, possibly mediated by inflammatory immune responses [137].

The autonomic nervous system regulation has also been suggested to potentially influence AMS development. Accordingly, excessively increased parasympathetic or sympathetic activity have been linked to the development of AMS [25,51,83,138] and epinephrine and norepinephrine levels are correlated with AMS severity [83]. Heart rate variability, as a marker of the autonomic nervous system, was characterized by a smaller variance in 32 adult on the way to an altitude of 3440 m [139]. An unfavorable heart rate variability increased the odds to develop AMS at 3440 m 7-fold in this study. Furthermore, another study found that changes in heart rate variability at 2400 m were related to AMS development when ascending to 3000-4300 m [140]. However, another study reported a lack of association between heart rate variability changes and AMS development [141], casting doubt on the suitability of heart rate variability as a prognostic marker for AMS.

Aside from physiological parameters, psychological factors are increasingly acknowledged as relevant contributors to successful high-altitude sojourns, yet they remain rarely explored [116,142,143]. The modulation of the brain and neurotransmitters by hypoxia (see section 4.6) may influence cognitive, anxiety and stress responses and help explain links with AMS. Specifically, psychological as well as hypoxic stressors activate both the sympatho-adrenomedullary system and the HPA axis, as previously reviewed [116]. Accordingly, a bidirectional amplification between psychologically and hypoxia-triggered physiological stress mechanisms seems likely. Accordingly, several studies reported increased cortisol levels during high-altitude exposure [144,145], sometimes higher in AMS [146]. However, the evidence is ambiguous. In some studies, neither consistent changes nor a robust link to AMS were found [147,148]. In one study, cortisol levels initially decreased at altitude, followed by a later rise above 5000 m [147]. Importantly, measurement variability, circadian fluctuations, and additional stressors such as exertion, weather, or unfamiliar situations influence cortisol independently of altitude and thus hamper comparison of studies [147].

Several previous studies investigated stress- and anxiety-related markers or mechanisms related to AMS [32,137,142]. Both hypobaric and normobaric studies have investigated anxiety in relation to AMS using self-report questionnaires such as the State-Trait Anxiety Inventory (STAI) to assess state (current) and trait (general) anxiety, or the Self-Rating Anxiety Scale (SAS) [142,149,150]. The physical symptoms of anxiety overlap with those of AMS, for instance, dyspnea, hyperventilation, and increased heart rate are typical of both anxiety reactions and significant altitude exposure [116]. This may suggest a bidirectional relationship, whereby state anxiety and altitude-induced symptoms can mutually reinforce each other [142,151], while at the same time complicating differential diagnosis [137]. Previous study results are heterogeneous. Several studies have reported an association between anxiety and AMS [137,[152], [153], [154]]. Oliver et al. further observed that while anxiety correlated with current symptom burden, it did not predict AMS on the following day [137]. Instead, upper respiratory symptoms - assessed with the seven-item modified Jackson scale (malaise, chilliness, sneezing, sore throat, runny nose, nasal congestion, cough) - as well as higher heart rate, lower peripheral oxygen saturation and reduced fluid intake emerged as predictors. More severe AMS symptoms were also associated with poorer physical and mental health, higher heart rate, and lower fluid intake [137]. Across multiple altitude levels, higher state anxiety (STAI-Y-1) correlated with higher AMS scores and lower peripheral oxygen saturation, suggesting that daily resting peripheral oxygen saturation may serve as an early indicator [137].

One report suggests that higher trait anxiety (STAI-Y-2) at low altitude predicts the subsequent development of severe AMS [142]. State anxiety (STAI-Y-1) did not differ at baseline but increased during ascent in parallel with AMS scores and correlated inversely with the peripheral oxygen saturation [142]. Elevated state anxiety (STAI-Y-1 >39) was more frequent in women and younger participants, in those assigned to earlier trekking teams (i.e., groups starting earlier in the day), and was associated with higher heart rate and worse AMS severity [142]. Nevertheless, the potential link regarding trait anxiety and AMS risk remains debated, possibly due to methodological differences between studies, altitude, type of hypoxia (hypobaric versus normobaric), population, or varying ascent rates [150]. In normobaric hypoxia (corresponding to 4500 m), no significant association between trait anxiety and AMS risk was observed within the total sample of 29 participants [149].

Overall, the relationship between AMS and psychiatric symptoms is still under-researched [155]. Besides the possible role of higher levels of anxiety prior to high-altitude exposure in AMS, also higher somatization score at sea level might be a predicting factor [156]. Moreover, depression and post-traumatic stress disorder present potential risk factors [151]. In summary, pre-existing psychiatric conditions may facilitate development and severity of AMS [142,154,157]. Moreover, overlaps of somatic and psychiatric symptoms (e.g., insomnia, tachycardia, hyperventilation) can complicate the differential diagnosis of AMS and psychiatric conditions and indicate the importance of clear guidelines for people with pre-existing psychiatric health conditions at high altitude [158].

**Fig. 2:**
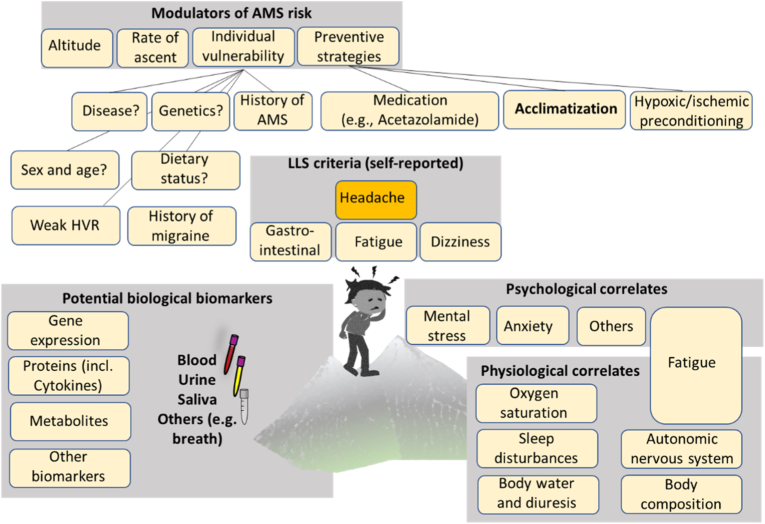
**Diagnostic criteria, correlates and potential biomarkers in acute mountain sickness (AMS).***HVR: hypoxic ventilatory response; LLS: Lake Louise Score*. *The figure was created with MS powerpoint.*

A promising approach to obtain predictive and diagnostic biomarkers is the combination of selected molecular biomarkers and physiological/psychological correlates of AMS, which is facilitated by the increasingly powerful computational possibilities for the analysis of multifactorial data. In a recent approach, Li and colleagues [36] used multidimensional phenotypic data and a machine learning approach (mutual information-radial kernel-based support vector machine-recursive feature elimination) to identify a set of two clinical features (systolic blood pressure and peak expiratory flow) and potential molecular biomarkers (6 proteins and 4 metabolites, see Table 2 for details) to predict AMS. A huge array of potential molecular biomarkers and physiological/psychological correlates is available to derive integrative models, taking into account a large number of potential risk factors (see Fig. 2).

However, it should be noted that a major drawback of correlation analyses in high-altitude field studies is their susceptibility to confounding variables that may obscure true associations. Factors such as physical exertion, nutritional status, hygiene-related challenges, and the prevalence of infections can all influence outcomes, potentially biasing results and limiting the reliability of the observed correlations. In addition, the level of altitude, speed of ascent and pre-acclimatization are strong modulators of AMS risk and must be taken into account.

## 6 Conclusions and perspective

Many published studies reported altered gene expression and protein levels following altitude exposure and the development of AMS (Table 1, Table 2, Table 3). Specific differences might be associated with protection from AMS, predict AMS or could eventually be used as biological diagnostic criteria for AMS. Although a few studies used validation cohorts to confirm findings on biomarkers from discovery cohorts obtained from the same group [37 84]or training and validation cohorts at real and/or simulated altitude [71], currently no biomarkers have been validated across different groups and/or are in routine clinical use. Our results suggest that (1) people respond very individually to hypoxia/altitude (which furthermore is certainly influenced by health and fitness status, biorhythms, diet, exertion/exercise and others) and/or (2) changes in the levels of specific markers/correlates are not sufficient to capture the complex pathophysiological changes involved in AMS and/or (3) different types of AMS exist that complicate the identification of reliable biomarkers. Moreover, biomarker analyses have been performed primarily in specific populations, for example transcriptomics-based studies were mainly performed in male Chinese lowlanders (Table 1), often limiting generalization to other populations.

One important open question relates to the general pathogenic processes underlying AMS. While inadequate hypoxia responses are most probably involved in the development of AMS, it remains debatable, if these responses are insufficient or, conversely, excessive. Based on the assumption that AMS can be caused, triggered or facilitated by a heterogeneous set of conditions, and that some individuals may be largely protected, while others are highly susceptible under these conditions to develop AMS, we support the view that different subtypes of AMS probably exist. Berger and colleagues previously suggested the possibility that subtypes of AMS can be differentiated, primarily based on the time course of symptoms [51]. These authors attributed AMS occurring during the first day (AMS type I) to factors like exertion, parasympathetic overactivity or masking by AMS-medications, while the later occurring AMS types II and III may be due to insufficient compensatory responses or even subclinical pulmonary clinical edema, respectively. Based on the reported potential biomarkers for AMS summarized in the present review, we assume that either weak hypoxia responses or overshooting hypoxia responses may be underlying mechanisms, which would be compatible with the suggestion of Berger and colleagues [51]. Evidence exists for both assumptions (insufficient versus excessive hypoxia responses), making it likely that AMS, as currently defined, can be caused through different mechanisms. An excessive hypoxia response as a potential cause for AMS is for example supported by higher VEGF levels in the blood of some individuals susceptible for AMS [38] or by stronger induction of antioxidant enzymes [81]. These overshooting responses could lead to maladaptive effects, involving for example excessive cerebral blood flow upregulation and/or blood-brain barrier disruption, associated with inflammatory and damage signaling.

Conversely, smaller hypoxic ventilatory responses in some people susceptible to AMS [6], or indications of reduced bicarbonate excretion [77] support a role of insufficient hypoxia responses in the development of AMS. Such moderate hypoxia responses may not adequately address the metabolic stress and energetic crisis caused by hypoxia/altitude exposure and thereby lead to AMS, for example by changes in neurotransmitter signaling.

**Fig. 3:**
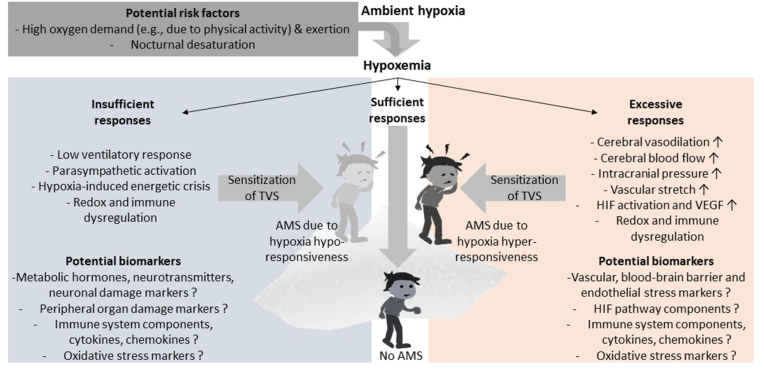
**Speculative model of acute mountain sickness (AMS) development through insufficient or excessive hypoxia responses.** If ambient hypoxia results in hypoxemia this usually results in hypoxia responses protecting from low oxygen availability in tissues. Hypoxemia can be facilitated by additional factors like high oxygen demand or pronounced nocturnal desaturation. We hypothesize that both insufficient and excessive hypoxia responses can promote the pathogenesis of AMS. It is possible that both pathogenic pathways can be characterized by specific sets of biomarkers but this needs experimental verification. *TVS: trigeminovascular system*. *The figure was created with MS powerpoint.*

Therefore, integrative and interdisciplinary approaches, considering various molecular, physiological and psychological aspects, in the future may help to identify personalized sets of biomarkers/correlates of AMS with high sensitivity/specificity. They should primarily be used to investigate the possibility to distinguish potentially different types of AMS, which may be a prerequisite to identify reliable and valid AMS biomarkers, predict and prevent AMS. If different types of AMS with distinct pathogenetic mechanisms exist, this could account for the lack of consistent AMS biomarkers until now. The possibility that AMS results from different extents (insufficient or excessive) of (patho-) physiological hypoxia responses could, for example, explain the high cardiovascular correlates of AMS in some susceptible individuals, while the cardiovascular responses of others appear to be less affected by AMS. Insufficient hypoxia responses may result in greater hypoxic stress in oxygen-dependent tissues possibly leading to energy homeostasis disruption in sensitive organs, a dysbalance in redox and immune regulation and inadequate inflammatory and oxidative stress responses, which in turn can sensitize the trigeminovascular system and trigger AMS symptoms. In contrast, excessive hypoxia responses may involve strong HIF-pathway activation resulting in pronounced upregulation of VEGF and also inadequate inflammatory and oxidative stress responses. In combination with cerebral vasodilation and blood flow, this also can sensitize the trigeminovascular system and trigger AMS symptoms. The involved processes may provide clues for AMS-type specific biomarkers but require experimental validation: a speculative model is shown in Fig. 3.

In addition (subclinical) vulnerabilities of specific organs (e.g., heart or kidney), may trigger forms of AMS with differential disease courses. This possibility could open up a novel approach to screening for organ vulnerabilities under hypoxic stress, potentially offering greater sensitivity than traditional screening methods.

High-throughput analysis techniques, including OMICs techniques, and ever more powerful, e.g., artificial intelligence-assisted, data analysis techniques enable the identification and systematization of large arrays of biomarkers. As highlighted in the present review, biological parameters, such as mRNAs, components of energy and lipid metabolism, inflammatory factors and many other signaling molecules and proteomic/metabolomic pattern changes have been associated with AMS. However, the results are largely inconsistent across studies and reliable sets of biomarkers are still lacking.

To advance the knowledge of AMS pathophysiology and enable the definition of clear biomarker sets, in our opinion two practical changes in the approach of AMS research are necessary. First, the traditional view that AMS represents one clearly definable disease entity should be replaced with the perspective that different individual predispositions and conditions can produce biologically and pathophysiologically diverse disease courses and subtypes, highlighting the need for personalization. Relevant individual predispositions include the individual hypoxic ventilatory response, insufficient versus excessive high-altitude responses and specific organ vulnerabilities. Important conditions comprise altitude levels, speed of ascent and temporal characteristics of AMS symptom development in the sense of Berger and colleagues [51].

Second, a holistic view combining molecular markers with physiological and psychological markers may be required to describe the potential subtypes and allow differential prediction, prevention and diagnosis. Consideration of population, environmental and ascent characteristics are essential, since they all may modulate the disease course.

## Funding

This research was funded in part by the Austrian Science Fund [Grant DOI: 10.55776/PAT6749924]. For open access purposes, the author has applied a CC BY public copyright license to any author accepted manuscript version arising from this submission.

## CRediT authorship contribution statement

**Johannes Burtscher:** Conceptualization, Investigation, Methodology, Project administration, Supervision, Visualization, Writing – original draft, Writing – review & editing. **Roxana Ehlers:** Writing – original draft, Writing – review & editing. **Nicolas Bourdillon:** Writing – original draft, Writing – review & editing. **Evelyn R. Pircher Nöckler:** Writing – original draft, Writing – review & editing. **Hannes Gatterer:** Writing – original draft, Writing – review & editing. **Katharina Hüfner:** Funding acquisition, Writing – review & editing. **Johanna M. Gostner:** Conceptualization, Supervision, Writing – review & editing.

## Declaration of competing interest

The authors declare that they have no known competing financial interests or personal relationships that could have appeared to influence the work reported in this paper.

## Data Availability

No data was used for the research described in the article.
